# Mutation screening of the *USH2A* gene in retinitis pigmentosa and USHER patients in a Han Chinese population

**DOI:** 10.1038/s41433-018-0130-3

**Published:** 2018-06-13

**Authors:** Lulin Huang, Yao Mao, Jiyun Yang, Yuanfeng Li, Yang Li, Zhenglin Yang

**Affiliations:** 1 Sichuan Provincial Key Laboratory for Human Disease Gene Study and the Department of Laboratory Medicine and School of Medicine, Sichuan Academy of Medical Sciences & Sichuan Provincial People’s Hospital, University of Electronic Science and Technology of China, 32 the First Ring Road West 2, Chengdu, Sichuan 610072 China; 2 Institute of Chengdu Biology, and Sichuan Translational Medicine Hospital, Chinese Academy of Sciences, Chengdu, Sichuan China; 3 Center of Information in Biomedicine, University of Electronic Science and Technology of China, Chengdu, Sichuan China; 4 Department of Ophthalmology, Beijing Tongren Hospital, Capital Medical University, Beijing, China

## Abstract

**Objectives:**

*USH2A* encodes for usherin, a basement membrane protein in the inner ear and retina. *USH2A* can cause retinitis pigmentosa (RP) with or without hearing loss. The aim of this study was to detect *USH2A* mutations in a Chinese cohort of 75 small RP families and 10 Usher syndrome families.

**Methods:**

We performed a direct Sanger sequencing analysis of the *USH2A* gene to identify mutations for this cohort.

**Results:**

We identified a total of eight mutations in four of the 75 small RP families (5.3%) and two mutations in one of the 10 Usher families (10%); all families were detected to have compound heterozygous mutations. In families with nonsyndromic RP, we identified the compound heterozygous mutations p.Pro4818Leuand p.Leu2395Hisfs*19 in family No. 19114, p.Arg4493His and p.His1677Glnfs*15 in family No.19162, c.8559-2A > G and p.Arg1549* in family No.19123 and p.Ser5060Pro and p.Arg34Leufs*41 in family No.19178. We also identified the heterozygous mutations p.Arg3719His and p.Cys934Trp in family No.19124, which was the Usher syndrome family. These mutations were predicted to be harmful by SIFT, PROVEAN, Mutation Taster or PolyPhen-2.

**Conclusions:**

Our results revealed six novel mutations in the *USH2A* gene in a Chinese population, which is beneficial for the clinical use of genetic testing of *USH2A* in patients with autosomal-recessive or sporadic RP and Usher syndrome.

## Introduction

Retinitis pigmentosa (RP, OMIM 226800) is one of the most common incurable eye diseases [1, 2] and has clinical and genetic heterogeneity [3, 4]. The worldwide prevalence of RP is 1:3,000 to 1:7,000 [5, 6]. Due to defects of the photoreceptor rods, the typical clinical symptom of RP is peripheral visual field loss, then gradual central vision loss, which may eventually cause blindness [5, 6]. RP can be inherited as autosomal dominant (30–40%), autosomal recessive (50–60%), or X-linked (5–15%) [5, 6]. So far, 87 genes have been identified as responsible for RP (https://sph.uth.edu/retnet/sum-dis.htm), including 27 genes for autosomal dominant RP, 57 genes for autosomal recessive RP, and three genes for X-linked RP. Most patients have nonsyndromic RP, while others suffer from associated syndromes. The most common syndromic RP is Usher syndrome, which accompanies RP with hearing loss in autosomal recessive inheritance, with a prevalence of approximately 1:20,000 [7]. Thirteen genes have been identified that may cause Usher syndrome (https://sph.uth.edu/retnet/sum-dis.htm).

The *USH2A* gene is located in chromosome 1 with 72 exons [8]. *USH2A* causes 10–15% of recessive RP cases and 30–40% of Usher syndrome type 2 cases. *USH2A* was first mapped in chr1q in Usher families by Lewis [9] and Kimberling in 1990 [10] and the latter identified *USH2A* as the candidate gene of Usher syndrome type 2in 1995 for the first time [11]. In 2000, Rivolta et al. identified for the first time missense mutations in the *USH2A* gene with recessive RP without hearing loss [12], suggesting that mutations in *USH2A* can cause Usher syndrome or RP only. In this study, we sequenced 75 RP and 10 Usher small families by direct Sanger sequencing of the exons of *USH2A*. We identified six novel mutations in the *USH2A* gene causing nonsyndromic RP or Usher syndrome in a Chinese population; this finding provides a theoretical basis for follow-up research and treatment of this disease.

## Methods

### Patient recruitment and ethics statement

This study was approved by the institutional review boards of Beijing Tongren Hospital and Sichuan Provincial People’s Hospital. All experiments were performed in accordance with relevant guidelines and regulations, including any relevant details. All of the participants received and signed the informed consent form. Seventy-five RP and 10 Usher small families were recruited from 2008 to 2012 from the clinicsoftheEye Center of Beijing Tongren Hospital, Capital Medical University.

The diagnosis of nonsyndromic RP was made in all individuals on the basis of an ophthalmologic examination, including visual testing, a slit-lamp examination, ophthalmoscopy, fundus photography, and electroretinogram (ERG). Patients with Usher syndrome reported hearing loss, which was tested clinically and verified by audiometry when required. Clinical data on each patient were included in this analysis. Venous blood (5 ml) was collected from each participant for DNA extraction with a GentraPuregene Blood DNA kit (China) according to the Gentra® Puregene® Handbook.

### Mutation screening

Mutation screening was performed by using the direct Sanger sequencing analysis. The coding region (exons 2–72) of *USH2A* was amplified by polymerase chain reaction (PCR) in these 75 families. The primers for the PCR amplification were previously reported 13. The coding regions (exons 2–72), including the intron–exon boundary of *USH2A*, were amplified by polymerase chain reaction (PCR). The amplification of target DNA by the PCR produces copies which may contain errors. To decrease the PCR/sequencing errors, we used pfu DNA polymerase to amplify the regions (PrimeSTAR^®^ HS DNA Polymerase with GC Buffer, KAKARA). High GC buffer was used when amplifying the high GC regions. We used forward and reverse primers to sequence them with the BigDye Terminator v3.1 Cycle Sequencing Kit (ABI Applied Biosystems) according to the manufacturer’s instructions and sequenced by ABI 3730 sequencer. Variant-filtering was based on public and in-house SNP databases, including dbSNP137, 1000Genome project, and ExAC Browser (Beta) Exome Aggregation Consortium data, as well as our internal database. We kept nonsynonymous and splicing variants with MAF < 0.1% for further analysis. The likely inheritance pattern of the genes, the functional impact, and the clinical relevance were analyzed to obtain possible mutations. The functional impact of each variant was predicted by SIFT and PROVEAN tools (http://sift.jcvi.org/), Mutation Taster(http://www.mutationtaster.org/) and PolyPhen-2(http://genetics.bwh.harvard.edu/pph2/PolyPhen-2). For each patient, a deleterious or damaged variant that was observed by at least one prediction tool was considered a mutation. The potential pathogenicities of the filtered variants were then interpreted according to the existing and proposed guidelines from the Standards and Guidelines for the Interpretation of Sequence Variants, to establish a diagnosis of the clinician made the final determination of the relationship of the reported variant(s) to the patient’s phenotype.

## Results

### Clinical characteristics of patients

**Fig. 1:**
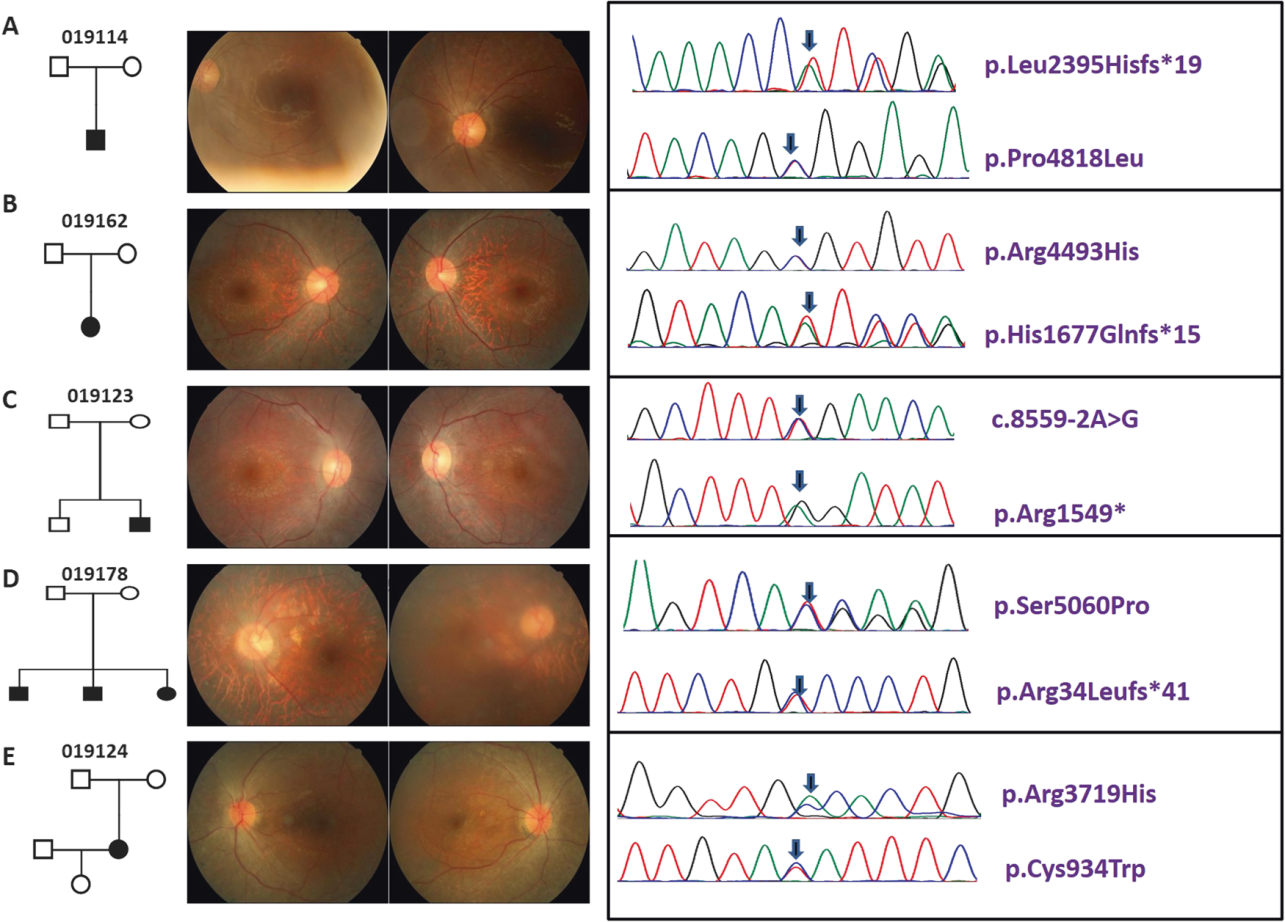
Pedigree, fundus pictures and chromatograms of detected *USH2A* mutations. **a** Pedigree, fundus pictures and chromatograms of detected *USH2A* mutations in 19114. **b** Pedigree, fundus pictures and chromatograms of detected *USH2A* mutations in 19162. **c** Pedigree, fundus pictures and chromatograms of detected *USH2A* mutations in 19123. **d** Pedigree, fundus pictures and chromatograms of detected *USH2A* mutations in 19178. **e** Pedigree, fundus pictures and chromatograms of detected *USH2A* mutations in 19124

The clinical information of the five patients is shown in Table 1. The pedigrees and fundus photographs of the five patients identified with have*USH2A* mutations are shown in the left and middle panels of Fig. 1. Patient No.19114 had suffered from night blindness since the age of 30; fundus photography displayed fundus pigmentation, and electroretinography (ERG) displayed no amplitude reactionPatient No.19162 had night blindness in both eyes since age 13; fundus photography displayed retinal pigment epithelial (RPE) cell-layer atrophy and vascular narrows, especially in the peripheral retina. There was no ERG reaction. Patient No. 19123 had night blindness since childhood; fundus photography showed a blue-greyperipheral retina, pigmentation, and vision loss. No. 19178 had night blindness since junior high school and was subsequently diagnosed with RP. Fundus photography displayed a concave-shaped reflective area in the central macula and black pigmentation in the posterior pole of the fundus. There was no ERG reaction. Patient No. 19124 had progressively declining vision, with night blindness and hearing abnormalities since age 42. Fundus photography displayed peripheral retinal turbidity and pigmentation resembled the appearance of bone cells. ERG displayed no amplitude reaction, and the patient was diagnosed with Usher-2 syndrome (Table 1).

**Table 1:** The clinical information of the five patients

	Age of onset	Gender	Diagnosis	Inheritance pattern	Family history	VA	Symptom	ERG
19114	30	M	RP	S	NO	0.8/0.5	Night blindness, pigmentation	Extinct
19162	13	M	RP	S	NO	0.1/0.3	Night blindness, pigmentation, macular convex reflective	Extinct
19123	7	M	RP	S	NO	0.5/0.8	Night blindness, pigmentation, retina gray	Extinct
19178	42	M	RP	S	Grandfather, brother, sister	0.01/0.1	Night blindness, pigmentation, optic yellow, RPE layer atrophy	Extinct
19124	42	F	Ush-2	S-USH	NO	0.8/0.7	Night blindness, hearing loss, pigmentation, peripheral retinal filth, 10^0^Tube video	Extinct

### *USH2A* mutation identification

All the variants detected in this study are listed in the Supplementary data. Totally, 1574 variants were detected. After variant-calling and data-filtering, we identified 10 compound mutations in five patients (Table 2), which contained nonsynonymous substitution, splicing, stopgain, frameshift deletion, and frameshift insertion. Four mutations were reported and six were novel. Patient No.19114 had nonsynonymous mutation p.Pro4818Leu (c.14453 C > T) [13] in exon 66 and the frameshift deletion p.Leu2395Hisfs*19 (c. 7183 _7193del) mutation in exon 38 (Table 1 and Table 2). His normal parents carried only one of these two mutations each. Patient No. 019162 had frameshift deletion p.His1677Glnfs*15 (c.5027_5043del) in exon 25 and a missense mutation p.Arg4493His (c.13478 G > A) in exon 63. Patient No. 19123 had a splicing mutant in exon 44 (c. 8559-2 A > G) and a stopgain mutation in exon 22 atArg1549* (c.4645 C > T) [8]. His normal brother only carried the splicing mutation. Patient No.19178 had frameshift insertion mutation p.Arg34Leufs*41(c.100_101insT) [14] in exon 2 and a missense mutation p.Ser5060Pro (c.15178 T > C) in exon70. His brother and sister also suffered from this disease, with the same mutations. The Usher-2 patient, No.19124, carried two missense mutations: p.Cys934Trp (c.2802 T > G) in exon13 and p.Arg3719His (c.11156 G > A) in exon57. Her parents carried one of the heterozygous mutations each, and her daughter wasnormal, carrying neither of these mutations.

**Table 2:** Mutation information of USH2A detected in this study

Family	Mutation			Source	PROVEAN	SIFT	Mutation Taster	PolyPhen-2
	Nucleotide change	Protein change	Domain					
19114	c.14453C > T	p.Pro4818Leu	NO	Aller, E. et al	Deleterious	Damaging	Might be affected	Probably damaging
	c.7184_7194del	p.Leu2395Hisfs*19	FN3	novel	NA	NA	Might be affected	Probably damaging
19162	c.5031_5047del	p.His1677Glnfs*15	LamG	novel	NA	NA	Disease casing	Probably damaging
	c.13478 G > A	p.Arg4493His	FN3	Novel	Neutral	Tolerated	Polymorphism	Benign
19123	c.4645 C > T	p.Arg1549*	LamG	Baux, D. et al.	NA	NA	Disease casing	NA
	c. 8559-2 A > G	p.Asp3515Gly	FN3	Nakanishi, H. et al.	NA	NA	Disease casing	NA
19178	c.100_101insT	p.Arg34Leufs*41	NO	Dai, H. et al.	NA	NA	Disease casing	Probably damaging
	c.15178 T > C	p.Ser5060Pro	Transmembrane region	Novel	Neutral	Tolerated	Polymorphism	Probably damaging
19124	c.2802 T > G	p.Cys934Trp	EGF_Lam	Novel	Deleterious	Damaging	Disease casing	Probably damaging
	c.11156 G > A	p.Arg3719His	FN3	Novel	Deleterious	Damaging	Disease casing	Probably damaging

### Analysis of harmful *USH2A* mutations by prediction

**Fig. 2:**
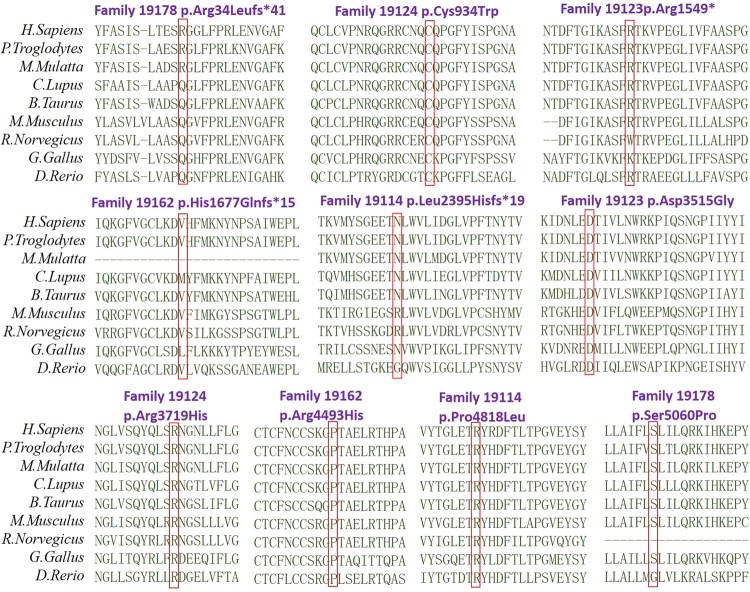
Amino acid mutations loci conservative in 8 species. Orthologous alignments of the detected mutations in this study suggest their evolutionarily conservative feature

To determine if the 10 changes identified in the families were pathogenic or not, we performed several prediction analyses. First, we examined the location of the 10 changes along the long usherin isoform and identified most of them located within functional domains (Table 2). Second, we aligned *USH2A* sequences from different species for each of the changes, all of which were evolutionarily conserved (Fig. 2). Third, based on damage prediction by SIFT, PROVEAN, Mutation Taster, and PolyPhen-2, all of these changes can be predicted as harmful by at least one predictor (Table 2). Thus, the results suggested that the 10 mutations might cause the clinical phenotypes of the patients with RP or Usher syndrome type 2 cases.

## Discussion

Mutations in the *USH2A* gene are responsible for the majority of USH2 cases [15, 16]. In addition to typical USH2, a certain mutant allele of the *USH2A* gene was also found to cause nonsyndromic RP with lessor no hearing defects [12]. *USH2A* mutations were estimated to underlie approximately7% of all RP cases in North America [17]. A report on Spanish patients showed that mutations in *USH2A* with autosomal recessive RP had high prevalence and phenotypic variations [18]. However, there is no report focusing on *USH2A* mutations in large samples of nonsyndromic RP small families in a Chinese population. In this study, we identified five pairs of compound heterozygous mutations in the *USH2A* gene from 75 nonsyndromic RP patients (5.3%) and 10 Usher patients (10%) in a Chinese population by a direct Sanger sequencing analysis.

*USH2A* is a large gene with 72 exons, encoding the protein usherin with 5202 amino acids. In mammalian photoreceptors, usherin is localized to a spatially restricted membrane microdomain at the apical inner segment recess that wraps around the connecting cilia, corresponding to the periciliary ridge complex described in amphibian photoreceptors [19]. The targeted defect of the *USH2A* gene in mice leads to progressive photoreceptor degeneration and moderate but non-progressive hearing impairment, mimicking the visual and hearing deficits in patients with *USH2A* mutations, suggesting its essential role for the long-term maintenance of retinal photoreceptors and the development of cochlear hair cells [19]. In this study, most of the 10 mutations might be responsible for nonsyndromic RP or USH2 because these changes are located in the functional domains and are predicted to be harmful for the normal function of the protein (Table 2).

**Fig. 3:**
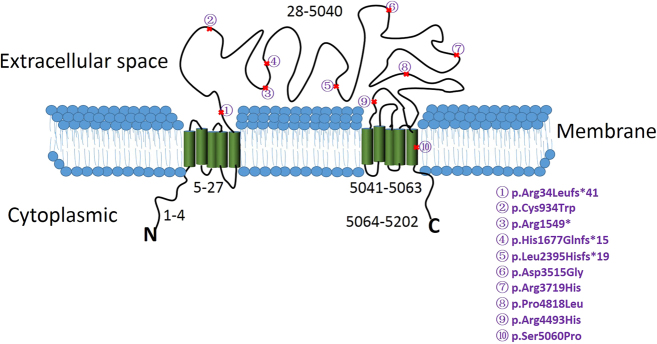
Predicted cellular distribution of the detected mutations.*USH2A* gene encodes a transmembrane protein. Mutation p.Ser5060Prois predicted in the membrane-bound protein, other mutations are distributed in the exocytoplasmic

Unexpectedly, in the comparison of the recorded mutations of *USH2A* in nonsyndromic RP to USH2 in the Human Gene Mutation Database (HGMD,http://www.hgmd.cf.ac.uk/ac/index.php), there was no obviously different distribution of these mutations between the two kinds of retinal diseases; the mutations are rather scattered, located in the whole protein. The mutations we detected in this study were located in exons 2, 7, 13, 22, 25, 38, 44, 60, 63, and 70; no more than two mutations were located in the same exon. Using the TMHMM2.0 software prediction for the usherin protein’s subcellular location, we found that nine of the 10 mutations could be located in the extracellular space (Fig. 3, subcellular location). Only one mutation(p.Ser5060Pro) is located in the transmembrane region. The usherin protein has a two-membrane structure domain in both the N and C terminals (Fig. 3, subcellular location). The extracellular space of the usherin protein may cooperate with other USHER proteins in the hair bundle of auditory sensory cells, and in the photoreceptor cells [20]. During the differentiation of the hair bundle, the extracellular space region of the usherin protein may be involved in the formation of a type of stereocilia side link located at the tip and the base of the stereocilia, contributing to the junction between the inner and outer segments, and at the synaptic region in the photoreceptor cells [20]. The defect in the usherin protein from congenital mutations may lead to the disorder of the connecting cilium in the photoreceptors, causing vision loss.

So far in the HGMD, most of the mutations have been identified in USH2 patients, while <10 mutations in nonsyndromic RP have been identified. In this study, we identified eight compound mutations in the *USH2A* gene from 75 patients with nonsyndromic RP and two compound mutations in 10 USHER patients. Six of the mutations we detected are novel. Aside from the severe mutations such as splicing, stopgain, and inframe mutations, the missense mutations are also harmful by prediction. Our results provide valuable information not only for precise clinical gene testing for *USH2A* in Chinese populations with nonsyndromic RP and USH2, but also for future studies performed to understand the molecular mechanism of *USH2A* in human disease.

### Summary

#### What was known before

- The compound heterozygous mutations p. P4818L in family No. 019114 have been identified.
- The compound heterozygous mutationsc.8559-2A > G and p.R1549X in family No. 019123 have been identified.
- The compound heterozygous mutations p.R34fs in family No. 019178 have been identified.

#### What this study adds

- We identified the compound heterozygous mutations p.2395_2398del in family No. 019114.
- We identified the compound heterozygous p.R4493H and p.1676_1681del in family No. 019162.
- We identified the compound heterozygous p.S5060P in family No. 019178.
- We also identified the heterozygous mutations p. R3719H and p.C934W in family No. 019124, which was the Usher syndrome family.

## Electronic supplementary material

Supplementary Table
